# Effective Emoticon Suggestion Technique Based on Active Emotional Input Using Facial Expressions and Heart Rate Signals

**DOI:** 10.3390/s23094460

**Published:** 2023-05-03

**Authors:** Jesung Kim, Mincheol Kang, Bohun Seo, Jeongkyu Hong, Soontae Kim

**Affiliations:** 1 School of Computing, Korea Advanced Institute of Science and Technology (KAIST), 291, Daehak-ro, Yuseong-gu, Daejeon 34141, Republic of Korea; jesung.kim@kaist.ac.kr; 2 Samsung Advanced Institute of Technology, 130 Samsung-ro, Yeongtong-gu, Suwon-si 16678, Republic of Korea; minc.kang@samsung.com; 3 Korea Financial Telecommunications and Clearings Institute, 9, Jeongjail-ro 213 beon-gil, Bundang-gu, Seongnam-si 13415, Republic of Korea; bohoon3355@kftc.or.kr; 4 Department of Computer Engineering, Yeungnam University, 280, Daehak-ro, Gyeongsan-si 38541, Republic of Korea

**Keywords:** computer-mediated communication, emotion recognition, emoticon suggestion

## Abstract

The evolution of mobile communication technology has brought about significant changes in the way people communicate. However, the lack of nonverbal cues in computer-mediated communication can make the accurate interpretation of emotions difficult. This study proposes a novel approach for using emotions as active input in mobile systems. This approach combines psychological and neuroscientific principles to accurately and comprehensively assess an individual’s emotions for use as input in mobile systems. The proposed technique combines facial and heart rate information to recognize users’ five prime emotions, which can be implemented on mobile devices using a front camera and a heart rate sensor. A user evaluation was conducted to verify the efficacy and feasibility of the proposed technique, and the results showed that users could express emotions faster and more accurately, with average recognition accuracies of 90% and 82% for induced and intended emotional expression, respectively. The proposed technique has the potential to enhance the user experience and provide more personalized and dynamic interaction with mobile systems.

## 1. Introduction

The evolution of mobile communication technology has had a profound impact on interpersonal communication, revolutionizing the way people communicate. In the past, people were limited to face-to-face interactions or written correspondence when communicating, but with the advent of mobile devices, people can now communicate in real-time from anywhere, exchanging information and emotions. Emotions are conveyed through various forms of mobile communication such as text, emoticons, pictures, and voice, providing users with a vast range of ways to express their emotions. From simple text-based messages to more complex forms of expression such as voice messages and video calls, these forms of communication have enhanced the emotional range of mobile communication technology.

Recent research has shown that mobile communication technology is highly effective for conveying emotions. For example, a study conducted by Gantiva et al. [1] found that the use of mobile devices to communicate emotions can lead to physiological responses, such as changes in heart rate and skin conductance. This suggests that the emotional impact of mobile communication technology is significant and has the potential to improve the quality of our daily interactions. Furthermore, previous studies have demonstrated no significant difference between computer-mediated communication (CMC) and face-to-face (F2F) communication in terms of semantic transmission. This means that people can convey the same extent of information and ideas through both CMC and F2F communication [2]. However, it is important to note that CMC may lack the nonverbal cues that are present in F2F communication, such as facial expressions and body language, which can make an accurate interpretation of emotions more difficult. Therefore, it is crucial to explore new techniques and approaches for enhancing emotional expression and recognition in CMC.

Researchers have conducted extensive studies on various emotion recognition techniques to effectively express emotions in CMC [3,4,5]. Among these techniques, facial expression recognition is widely used in computer vision [3,6]. Another potential solution for recognizing emotions is using human speech, which has been investigated in several studies [7,8]. These techniques can improve recognition accuracy by utilizing efficient algorithms and applying new models. In addition, multiple modalities, such as facial expressions, human speech, and body gestures, can complement the weaknesses of each technique, leading to better overall recognition performance [9]. Recently, diverse physiological sensors for detecting emotions have been developed [4,10,11,12]. This approach involves capturing signals from the human body using these sensors and determining human emotions based on the different conditions of the physiological data. By analyzing physiological signals, such as heart rate and skin conductance, researchers can infer a person’s emotional state, and use this information for emotion recognition. The development of physiological sensors provides an additional modality for emotion recognition and has the potential to enhance the accuracy and robustness of emotion recognition systems. Although many proposed techniques have been studied to increase the performance of emotion recognition, only a few studies have examined the psychological aspect of emotion recognition. Because human emotions are highly coupled with psychological behavior, the psychological activity must be considered before studying them.

In this paper, we introduce a new approach for recognizing emotions in mobile environments, based on psychological and neuroscientific principles. According to psychology, there are two main reasons that people express emotions [13]. First, an emotional expression is an initial reaction to an external stimulus (known as the induced emotional expression). Second, emotional expressions can also result from more detailed information processing (known as intended emotional expression). This means that when evaluating emotion recognition techniques, it is important to consider not only the accuracy of the technique itself but also the purpose of the emotional expression. Facial information is commonly used for intended emotional expression, as it is considered an explicit form of emotional expression. For induced emotional expression, heart rate (HR) information is also captured [4,14]. Both types of information can be easily obtained in a mobile environment.

The system recognizes users’ primary emotions, including neutrality, happiness, sadness, and anger, through facial and HR-based emotion recognition techniques, which can be implemented using a front camera and an HR sensor, respectively [4,13,14]. Although the recognition rate of negative emotions is lower than that of positive emotions for facial emotion recognition, the use of HR-based techniques helps to augment the recognition of negative emotions, thus improving the overall accuracy [15]. By combining facial and HR-based emotion recognition, the proposed approach provides a more comprehensive and accurate assessment of an individual’s emotional state, which can serve as active input in mobile systems.

We conducted a user evaluation to verify the efficacy and feasibility of our proposed technique. Our evaluation results showed that, on average, users could express induced and intended emotions 14.26% and 16.22% faster, respectively. In addition, the average recognition accuracies were 90% and 82% for induced and intended emotional expression, respectively. However, a high accuracy recognition rate does not guarantee efficient and convenient emotional expression because it is affected not only by the recognition rate but also by the workload of the user. Hence, we performed the NASA-TLX workload evaluation to quantify the user-induced overall workload for the proposed technique.

The contributions presented in this study are as follows:

- Active Emotional Input (AEI): A user-centered approach to emotion recognition that encourages users to intentionally express their emotions.
- A design that incorporates psychological factors and usability concerns for a seamless and efficient user experience in computer-mediated communication environments.

The remainder of this paper is organized as follows. Section 2 presents previous studies on emoticon recommendation techniques. Section 3 provides a brief background to understand the proposed technique. In Section 4, we introduce the proposed emotion recognition technique and describe our emotional expression evaluation based on an open-source messenger, which was modified to incorporate facial and HR-based emotion recognition techniques. Section 5 presents the experimental results. In Section 6, we discuss these results in detail. Finally, we conclude the paper in Section 7.

## 2. Related Work

This section presents a survey of the relevant literature regarding emoticons and their role in affective communication, as well as exploring the latest developments in multi-modal emoticon suggestion techniques in mobile environments.

### 2.1. Emoticon Suggestion Systems

Several studies [16,17,18,19,20,21,22,23,24,25,26] have been dedicated to improving the efficiency of emoticon suggestions. One of these studies, conducted by Pohl et al. [17], proposed EmojiZoom, an innovative emoticon input method that surpasses the traditional emoticon keyboards based on long lists. Another study by Chen et al. [21] found intriguing evidence of a significant difference in emoticon usage between male and female users. Miller et al. [16] explored whether the rendering of emoticons or differences across platforms could lead to diverse interpretations of the same emoticon. This was further investigated through a survey of over 2000 participants by Miller et al. [18], who found that text could both increase and decrease the ambiguity of emoticons. Liebeskind et al. [23] examined highly sparse n-grams representations and denser character n-grams representations for emoticon classification. Chen et al. [24] used emoticon-powered representation learning for cross-lingual sentiment classification. The emotional components of emoticons [25] were also found to be crucial for comparing and contrasting the associations between emoticons and emotions across cultures. An attention mechanism [22] was employed to better understand the intricacies underlying emoticon prediction and select the most important contextual information [19]. Lastly, Cappallo et al. [20] predicted emoticons from both text and images and considered the challenge of accounting for new and unseen emoticons. [26] presented the Context-Aware Personalized Emoji Recommendation (CAPER) model, which combines contextual and personal information to make recommendations. The model outperformed existing methods, demonstrating the effectiveness of considering both contextual and personal factors.

### 2.2. Commercial Products for Emoticon Suggestion

Emoticon suggestion has become a popular feature in many products. While there are innovative new technologies such as Animoji/Memoji [27] on recent iPhones and ARemoji on Samsung Galaxy mobile phones that use a camera to recognize facial expressions and generate an animated emoticon, a simpler and more intuitive way of suggesting emoticons is through the "favorite emoticon list" available on most default mobile operating system keyboards. This list typically organizes emoticons based on their frequency of recent usage. Some advanced techniques can be found in various applications, such as Line [28], Google GBoard [29], TouchPal [30], Word Flow [31], and SwiftKey [32], which map specific emoticons to specific words. For example, when a user types the word “love”, the app suggests a relevant emoticon. Minuum Keyboard [33] offers model-based emoticon suggestions by considering the current text input of the user and recommending appropriate emoticons based on a suggestion model. However, these apps only suggest a limited number of emoticons, which restricts the user’s freedom to choose from a wider variety of options. Furthermore, as they only analyze the current text input, they may not consider the various contexts of the conversation and do not provide suggestions for emoticon-only sentences.

### 2.3. User Interfaces for Emoticons using Multi-Modal Signals

To the best of our knowledge, there have been limited studies on support for users to create and communicate through multi-modal emoticons. Nevertheless, the HCI community has produced and examined numerous innovative user interfaces aimed at enhancing communication through visual emoticons or multi-modal signals such as haptic feedback.

Research has been conducted to investigate new methods of communication through pictorial emoticons or emojis. For example, Opico [34] facilitates communication, enabling users to express feelings or simple concepts through sequences of emoticons. MojiBoard [35] simplifies the use of parametric emoticons to convey emphasis or short stories using a keyboard. Some studies have aimed to fully automate the process of selecting and sending emoticons, such as ReactionBot [36], which adds emoticons to text messages based on users’ facial expressions. Another study examined the face-to-emoticon concept [37]. The selection of emojis/emoticons has also been automated using emotion keywords [38], sentences [39], and speech signals [40]. Voice-based emoticon entry for visually impaired users was explored in Voicemoji [41]. Some studies have addressed challenges related to the accessibility and inclusiveness of emoticons [42]. Customization of emoticons by users, such as the creation of new emoticons based on sketches and text input, has received increased attention [43].

## 3. Background

This section describes the impact of emoticons on communication and their role in emotional expression. In addition, the significance of heart rate-based emotion recognition is presented.

### 3.1. Emoticons and Their Impact on Communication

Emoticons first appeared in computer systems in the 1970s in the PLATO system [44] by Fahman in 1982 [45]. While the terms “emoticon” and “emoji” are often used interchangeably, “emoji” originated in the Japanese mobile market and means “facial letters/characters” [44]. Emoticons and emojis have become a widely used pictographic language for expressing emotions, resulting in numerous studies on their usage in various contexts, such as in software developers’ communication [46], students’ learning [47], and people’s political attitudes [48]. Another area of research delves into the contextual and personal aspects of emoticon usage, such as re-purposing emojis beyond their original meaning [49], reducing dependence on text [50], and customizing usage [51,52]. The study of emoticons and emotional communication frequently relies on theories and models of emotions, including discrete [53] and dimensional emotion theories, such as Russel and Barrett’s valence-arousal model [54]. For example, Rodrigues et al. established the Lisbon Emoji and Emoticon Dataset (LEED) [55], which captures participants’ perceptions of 238 emoticons and the corresponding valence and arousal dimensions. These emotional properties of emoticons inform both the analysis of emoji usage and the creation of emotional interfaces. LEED was used in this study to develop a recommendation algorithm for multi-modal emoticons.

### 3.2. Role of Emotional Expression

In psychology, the expression of emotions is a complex and multifaceted phenomenon that can be divided into two main categories: induced emotional expression (IDE) and intended emotional expression (ITE) [13].

- **Induced emotional expression (IDE):** IDE refers to the initial assessment of an external stimulus. This type of emotional expression is often automatic and unconscious, and can be seen in behaviors such as smiling when feeling good [13,56].
- **Intended emotional expression (ITE):** ITE, on the other hand, involves more detailed information processing for intentional purposes. This type of emotional expression is often deliberate and controlled, and is used for social or communicative purposes, such as smiling to ingratiate oneself with others, regardless of one’s actual feelings [57,58].

Both IDE and ITE play important roles in the expression of emotions and provide valuable information about an individual’s emotional state. Understanding the distinction between these two types of emotional expression is crucial for accurately assessing and interpreting emotional expressions in various contexts.

These two aspects of emotional expression have important implications for the design of emotion recognition systems. When considering which properties of emotional expression should be given higher priority, it is important to note that transmitting emotions through facial expressions is a natural and common behavior in human communication [57,59]. As such, IDE, which is primarily captured through facial information, should be given priority in the design of an emotion recognition system.

The second aspect is which recognition techniques are appropriate for each emotion. Although cultural differences can play a role in emotional expression, evidence suggests that people tend to suppress negative emotions in similar ways [60]. This social characteristic can make it difficult to recognize negative emotions through explicit expressions, such as facial expressions. In such cases, it may be more effective to focus on detecting and recognizing changes within the body, such as changes in heart rate, to accurately assess negative emotions.

In conclusion, these two important aspects of emotional expression highlight the need to carefully consider the design of an emotion recognition system. By prioritizing IDE and recognizing the difficulties in detecting negative emotions through explicit expressions, a more accurate and effective system for emotion recognition can be developed.

### 3.3. Heart Rate-Based Emotion Recognition

**Figure 1:**
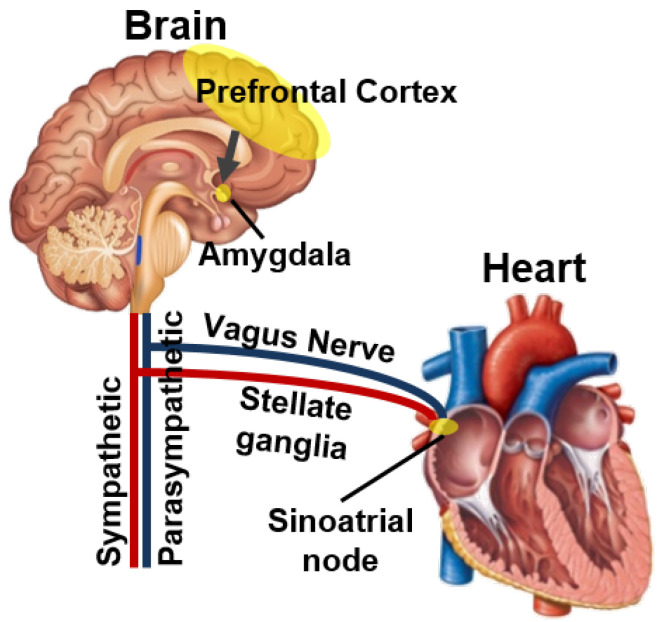
The connection structure between the nervous system and the heart.

Figure 1 depicts the connection structure between the nervous system and the heart. HR-based emotion recognition techniques offer a practical and accessible solution for incorporating emotions as active input in mobile systems. These techniques take advantage of the connection between brain activity and HR, which is monitored through the autonomic nervous system (ANS) [15]. The ANS is responsible for regulating unconscious bodily functions such as heart rate, blood pressure, and respiration.

The ANS is divided into two main components: the excitatory sympathetic nervous system (SNS) and the inhibitory parasympathetic nervous system (PNS). During negative emotions, the prefrontal cortex, a region in the brain responsible for executive functions such as decision-making, social behavior, and emotional regulation, inhibits the activity of the amygdala, leading to increased activity of the SNS. This increased SNS activity results in an increase in HR through the sinoatrial node and stellate ganglia [15]. Conversely, during positive emotions, the activated amygdala leads to increased activity of the PNS, causing a decrease in HR through the vagus nerve.

The fluctuations in HR, which are caused by neuronal activity, can be utilized to accurately evaluate an individual’s emotional state. However, it is crucial to acknowledge that these changes must occur within an appropriate time frame for the system to accurately recognize them. The ease of integrating HR-based emotion recognition techniques into mobile devices, such as smartphones or wearables [15], renders them a valuable solution for integrating emotions as active inputs in mobile systems.

In conclusion, HR-based emotion recognition techniques offer a practical and accessible solution for incorporating emotions as an active input in mobile systems. By taking advantage of the connection between brain activity and HR, these techniques provide a comprehensive and accurate assessment of an individual’s emotional state, making them valuable tools for enhancing the user experience in mobile systems.

## 4. Materials and Methods

In this section, we introduce our emotion recognition mechanism and active emotional input interface. Additionally, we provide details regarding the prototype implementation of the proposed solution.

### 4.1. Usability of Emoticons in Mobile Environment

In the field of human-computer interaction, a critical aspect is understanding the time required to reach a target in a graphical user interface [61]. The relationship between the size of the target and the distance to the target plays a significant role in determining the movement time required to reach the target.

This concept can also be applied to the design of emoticons for mobile messengers. The size and distance of emoticons can affect the time it takes for users to select the desired emoticon. By optimizing the size and placement of emoticons, the user experience can be improved by reducing the time and effort required to select emoticons. Additionally, the layout of emoticons can be optimized to minimize the movement distance between commonly used emoticons, further improving the efficiency and speed of user interactions.

**Figure 2:**
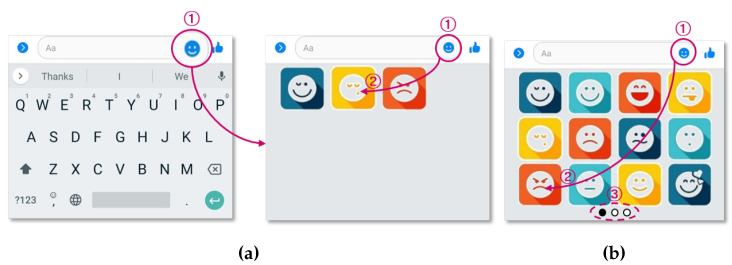
Examples of using emoticons on a mobile keyboard. (**a**) Basic arrangement of emoticons. (**b**) Extra emoticons.

Figure 2a shows a keyboard layout in a mobile environment. Touching (1) brings up the list of emoticons on the keyboard, and the user can touch the desired emoticons from the list. In this example, it is assumed that the emoticon located at (2) in Figure 2a is touched. Simply put, the more distant the emoticon to be used is from the icon that calls up the emoticon list, the greater the movement time. Figure 2b shows the appearance of the existing emoticon collection with several additional emoticons [27,28]. In this case, the potential distance between (1) and (2) increases, leading to an increase in movement time. If a large number of emoticons are added and the current keyboard cannot display their entire list, (3) is used to swipe the keyboard to output the next emoticon list. In this case, the potential movement time increases even further.

The greater the number of emoticons, the more difficult it becomes for the user to remember where the desired emoticon is located. As a result, additional time is consumed to recognize the desired emoticon from among the emoticons displayed on one screen. In this situation, a system that classifies and proposes the emoticon currently required by the user becomes an efficient means for the user to express emotions smoothly in a CMC environment.

**Figure 3:**
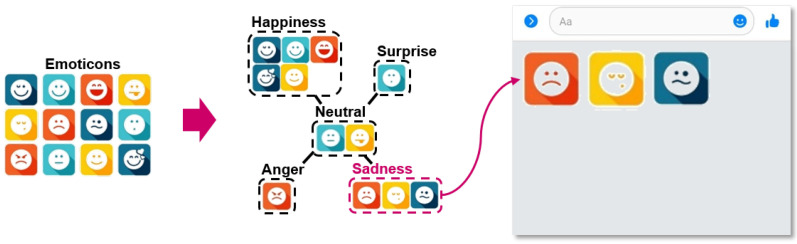
User emotion-based emoticon recommendation system.

Figure 3 illustrates the goal of the proposed technique. This technique filters out emoticons related to the emotions currently expressed by the user from among the unclassified emoticons and recommends them. Note that the proposed technique only targets emoticons related to emotions and does not apply to emoticons that are not related to emotions, such as food, objects, and animals. The user’s emotions are recognized and classified into five prime emotions: happiness, surprise, neutrality, sadness, and anger. Finally, only emoticons related to the recognized emotions are displayed on the keyboard layout.

As mentioned in Section 2, previous studies have proposed various techniques for recommending emoticons that match the current conversation’s context or recognize the user’s emotions in a non-intrusive manner. However, these techniques do not consider the psychological factors of users. As described in Section 3.2, human emotional expressions do not necessarily match the emotions they actually feel. For example, we may have to smile even when we are angry or remain expressionless even when we are happy. Therefore, a mechanism for recognizing the emotions that the user actually wants to express and recommending emoticons associated with these emotions is required.

### 4.2. Emotion Recognition Mechanism

Facial expressions are the primary way in which people naturally communicate their emotions in real-world interactions. These expressions reflect the user’s intentions and can be controlled at will. However, the various physical changes that occur when emotions are felt, excluding facial expressions, are mostly beyond our control. For example, changes in skin conductance, an increase in body temperature, and fluctuations in heart rate are difficult to intentionally control. Therefore, our proposed technique prioritizes facial expression recognition as the primary input for emotion recognition, which reflects the user’s intention and purpose. Based on this, the technique considers secondary physical changes that occur to recognize the user’s actual intended emotion. In a mobile environment, such as a smartphone, facial expressions can easily be captured using the front camera. Furthermore, recent wearable devices such as smartwatches are equipped with sensors such as heart rate sensors and electroencephalography (EEG) sensors, making it easy to measure a user’s various physical changes. However, to determine the user’s emotions through these various measured physical changes, standards are required.

The technical and emotional characteristics of the five prime emotions are outlined in Table 1. Emotions such as happiness and surprise can easily be detected simply by observing facial expressions. However, sadness can be difficult to recognize, as people tend to hide their negative emotions, as mentioned in Section 3.2. Distinguishing emotions such as neutrality and anger can also be challenging, as they correspond to similar facial expressions. However, these inaccuracies can be compensated for by monitoring HR variations. For instance, anger can be differentiated from neutrality by the higher HR that results. By giving more weight to sadness when HR remains elevated, the recognition of sadness can be improved. By combining facial expressions and HR information, the current emotional state can be more accurately recognized, as shown in Table 1. This integration enhances the accuracy of emotion recognition in the proposed technique, providing a more complete evaluation of an individual’s emotional state.

The proposed emotion recognition technique combines the use of facial expressions and HR information to provide a practical and effective way to incorporate emotions as active input in mobile systems. The unique characteristics of both facial expressions and HR are utilized to deliver a comprehensive and accurate assessment of an individual’s emotional state, making it a valuable asset for improving the user experience in mobile systems.

### 4.3. Active Emotional Input Interface

Our proposed technique prioritizes recognizing the emotions that the user actually wants to express. This approach opens up the possibility of using emotions as active input in systems such as mobile devices, rather than simply recognizing emotions. In more concrete terms, the users can actively express their emotions at the moment they desire through physical action, such as touching the screen with their finger or pressing a physical button, and using this expression as an input for the system. We refer to this input as “active emotional input (AEI)”. AEI allows the user to input one of the five prime emotions into the system through their emotions. This differs from traditional, non-intrusive emotion recognition methods in that the user must actually bring to mind a specific emotion to induce physical changes. This requires evaluations of how easily the user can evoke emotions and how quickly and accurately the system can recognize them.

Our proposed technique uses the front camera on a mobile device and a heart rate sensor to measure the user’s emotions, and the evaluation results are described in Section 5. The AEI was also evaluated using the same sensors. However, this does not mean that the AEI must be implemented through the camera and heart rate sensor. It can also be implemented using sensors, such as EEG sensors, that can complement facial expression recognition systems.

### 4.4. Prototype Implementation

**Figure 4:**
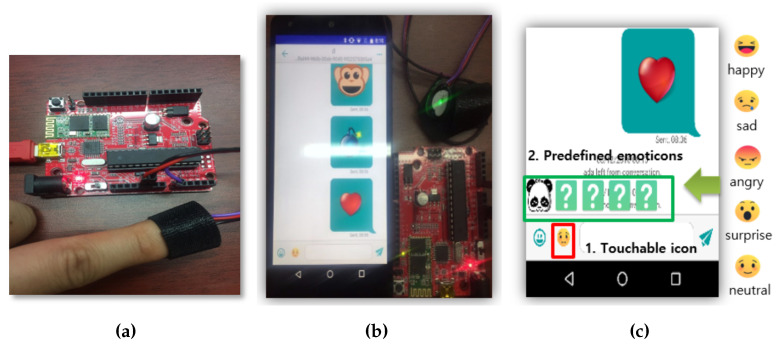
Implemented prototype sensor hardware and the messenger interface. (**a**) HR sensor device. (**b**) Device connection. (**c**) Messenger interface.

To evaluate the efficacy and feasibility of the proposed emotion recognition technique, we conducted experiments by applying it to an instant messenger. The physical prototype implementation of HR sensing is shown in Figure 4a. We added a finger-attached HR sensor to an Arduino to accurately measure the heartbeat once per second. Note that it is not necessary to use this type of sensor and various wearable devices equipped with heart rate and EEG sensors can be used. Figure 4b shows the mobile device and the heart rate sensor. The HR sensor transmits the user’s heartbeat, measured every second, to the mobile device through Bluetooth, and this data is transferred to the instant messenger and processed internally. The instant messenger used in this study is the SPIKA open-source instant messenger [62], which was modified to run on a Google NEXUS 5X device. The modified instant messenger also captures an image of the user’s face every second through the front camera of the device. These images are then transmitted to a facial emotion recognition algorithm [63] for processing.

As shown in Figure 4c, the recognized emotion can be viewed through the icon displayed at the bottom of the screen. The location marked in the red square (1) displays the current emotion recognized by the system, which can be happiness, sadness, anger, surprise, or neutrality. This allows the user to recognize the emotions perceived by the system before sending a predefined emoticon. Touching the icon displays predefined emoticons (2) at the top of the icon. It is assumed that each emoticon is associated with a specific emotion when registered in the system, and the proposed technique does not recognize the emotion represented by the emoticon.

**Figure 5:**
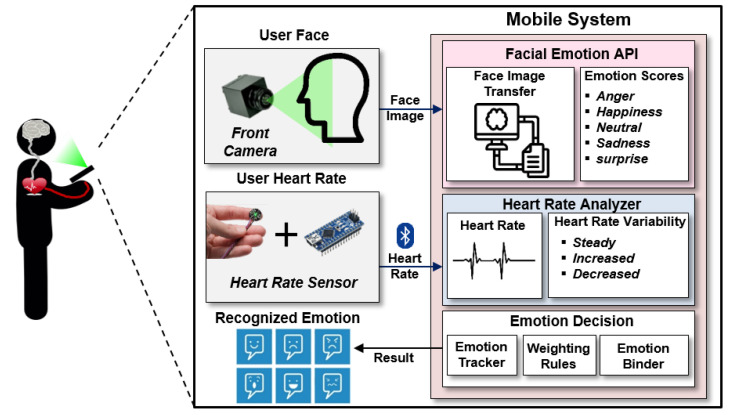
Proposed emotion recognition architecture.

Figure 5 illustrates the software architecture of the proposed technique within the instant messenger. The proposed software is composed of three parts: the Facial Emotion API, Heart Rate Analyzer, and Emotion Decision. The user’s face, which is received through the front camera of the mobile system, is used as input for the algorithm to recognize emotions. We used the Microsoft Face API [63] to recognize emotions through the face. The heart rate analyzer recognizes the user’s emotions based on the heart rate received every second, and we used our own in-house recognition algorithm. Emotion recognition based on the heart rate should be based on the previous state of the user, as the average heart rate and heart rate changes vary from user to user, unlike facial expressions.

This approach allows our proposed method to continuously calculate the probability of each of the five primary emotions at any given moment. Our modified messenger internally records users’ activities and the accuracy of emotion recognition in a quantitative manner. This internal structure enables us to precisely determine when users successfully express their emotions, allowing us to use the data calculated at those moments for our analysis.

## 5. User Study

In this section, we present the results of the user study that we conducted to evaluate the performance of our proposed emoticon suggestion technique.

### 5.1. Study Design

We evaluated the proposed technique to verify its efficacy and feasibility in terms of expressing emotions using emoticons. Specifically, we compared our emotion recognition mechanism with search and select (S&S), which is the conventional method most users currently use to select emoticons through touch and swipe gestures. We did not choose facial or heart rate-based techniques as our competitive methods, as the goal of our research is not to develop a technology with superior performance to previous related techniques. Eleven people of various occupations and ages participated in this experiment. We instructed all participants on how to use our modified messenger and proposed an emotion recognition mechanism. In addition, we asked each of them to wear an HR sensor on their finger.

We conducted an experiment to examine both induced emotion expression (IDE) and intended emotion expression (ITE) as described in Section 3.2 using two types of procedures, A and B. Participants were asked to complete both types in a randomized order. In Type A, participants watched 5-min video clips designed to evoke one of the five prime emotions: calmness, happiness, sadness, fear, and surprise. While viewing the videos, they were randomly prompted to express their current emotions using emoticons in a chat application, employing both familiar methods from other chat apps and the proposed method from our study. Following the emotion expression task, participants completed a 20-min survey related to the experiment. In Type B, participants started by maintaining a state of calmness until their heart rate stabilized. Once stable, they were asked to express each of the five prime emotions using emoticons provided in a chat application on a mobile device, again using both familiar methods and the proposed method. After completing the emotion expression task, participants filled out a 20-min survey related to the experiment. For Type B, participants were required to express emotions in a way that the system could recognize, such as smiling or making a surprised facial expression. The experiment was conducted as described, using randomly selected video clips as emotional stimuli to evoke specific emotions.

### 5.2. Emotion Recognition Accuracy

Table 2 shows recognition rates for each IDE and ITE scenario. Note that we did not measure the accuracy of S&S as it is always successful. The table represents the average scores of the recognized prime emotions through our proposed technique. For example, when the participants expressed happiness in the IDE scenario, the proposed technique determined that they had a 95% probability of happiness and a 5% probability of anger. The average recognition accuracies of 90% and 82% for induced and intended emotional expression, respectively.

It was shown that the accuracy was lower for ITE compared to IDE for each prime emotion except neutrality. In our qualitative evaluation, the participants found it relatively difficult to express a specific emotion voluntarily, excluding neutrality. For example, in the case of happiness, participants showed discomfort in smiling through the front camera, resulting in an accuracy of 95% for IDE and 72% for ITE. For the surprise emotion, accuracy improved by 4% for ITE compared to IDE. This is explained by two reasons. First, during the IDE scenario, participants did not clearly show an explicit expression of surprise when they were emotionally surprised and kept a somewhat neutral expression. Nonetheless, the proposed technique recognized the participants’ state as a surprise by capturing their sudden increase in heart rate. Second, in the ITE scenario, there was no change in heart rate, but the system was able to recognize the surprise state by the user explicitly making a surprised expression. This means that the proposed technique can accurately recognize the user’s emotions based on two complementary physiological features and successfully propose a related emoticon.

### 5.3. Completion Time for Expressing Emotions

**Figure 6:**
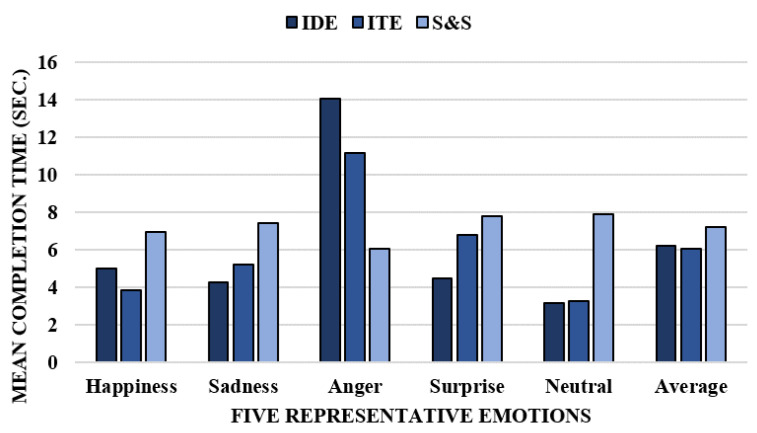
Completion time of expressing five prime emotions.

The mean completion time for expressing emotions is shown in Figure 6. Three types of tasks were included for each emotion: IDE, ITE, and S&S method. When a participant successfully completes their emotional expression, we record the probabilities assigned to each emotion by our emotion recognition technique at that specific point in time. The conventional procedure for expressing emotions involved searching for and selecting emotions, and in the conventional procedure of the evaluation, all emotions could be expressed in between 6 and 8 s.

Expressing happiness and anger had a shorter completion time in ITE than in IDE. Participants expressed induced happiness from the video context in ITE, as opposed to manipulated happiness in IDE. The non-continuity of induced happiness, which relies heavily on the video context, prevented participants from continuously expressing happiness, enabling the proposed emotion recognition mechanism to quickly and accurately recognize happiness. However, it was more natural for participants to express induced happiness in ITE. As a result, the completion time for expressing happiness was shortened in ITE than in IDE. Participants found it difficult to express anger in the IDE procedure, which highlights the difficulty in rapidly expressing manipulated anger. The recognition of anger is not solely based on facial emotion information, but also on the HR state. However, the external stimulus of anger from the video context in ITE helped participants express anger more efficiently, resulting in a shorter completion time for anger expression in ITE than in IDE.

Expressing sadness and surprise also had shorter completion times in ITE than in IDE. Participants did not seem to focus on facial expressions when expressing sadness, and the completion time of surprise differed between the intended and induced facial expressions. Intended or manipulated surprise needed to be explicit for others to perceive the expression, leading to a longer completion time for recognition in ITE. However, the actual surprise does not need to be explicit, and the time of expression is typically instant in a real-life surprise scenario, which requires a longer time to be recognized in ITE.

**Figure 7:**
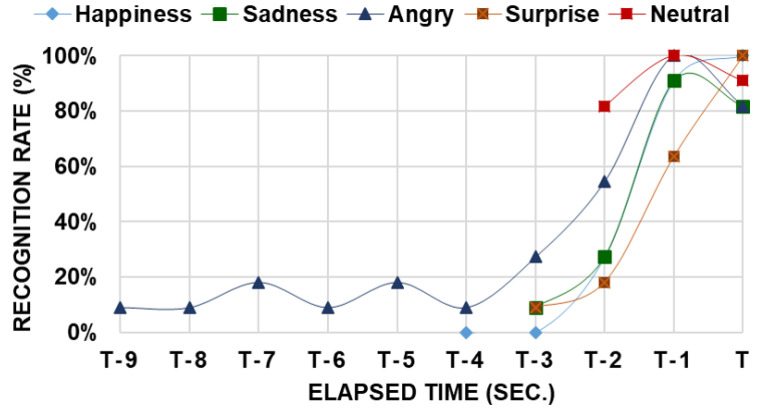
The change in recognition rate for prime emotions.

Figure 7 shows the variation in recognition rates for the five emotions using the results of the IDE procedure. The mean completion time for expressing each emotion was measured in seconds, with elapsed time T denoting the mean completion time. The variation in recognition rates for neutrality can be traced between T-3 and T, as the mean completion time for expressing neutrality was approximately 3, as shown in Figure 6. Similarly, the mean completion time for expressing surprise was approximately 4, and tracing was available between T-4 and T. The variation in recognition rates in the ITE procedure is similar to that in the IDE procedure.

Expressing anger had the longest mean completion time compared to the other emotions. Although anger had the longest mean completion time, its recognition rate did not increase linearly, as shown in Figure 7. The recognition rate of anger remained below 20% from T-9 to T-4, after which it rapidly increased. This phenomenon highlights that the changes in the HR state resulting from emotions require additional time to be reflected, despite the HR being controlled by the nervous system based on electronic signals. The time that elapsed before a rapid increase in the recognition rate is referred to as the emotional loading time. For anger, the emotional loading time was at least 5 s, as shown in Figure 7.

### 5.4. Task Workload Analysis for Expressing Emotions

**Figure 8:**
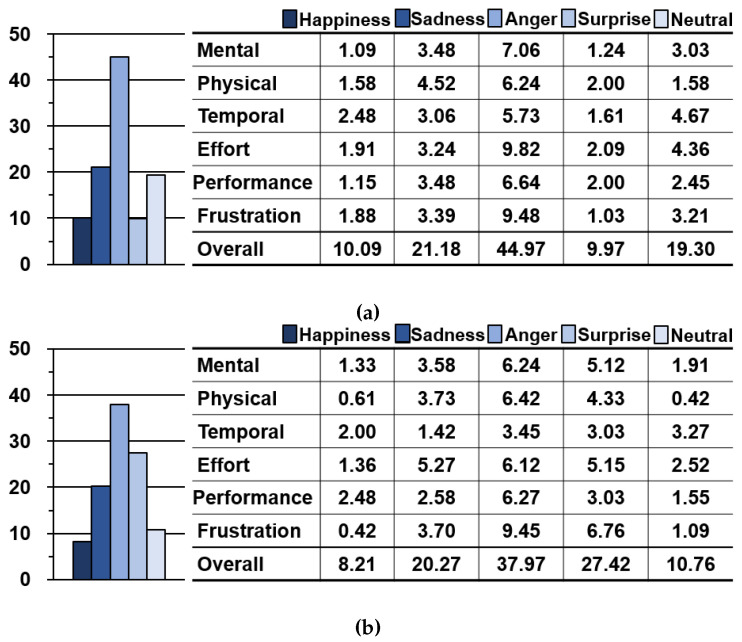
NASA-TLX workload results for representative emotions. (**a**) Results for IDE procedure. (**b**) Results for ITE procedure.

Figure 8 shows the NASA-TLX workload for expressing the representative emotions of our evaluation procedures, except for the conventional one.

The workload for expressing happiness and sadness is similar in both the IDE and ITE procedures, which require the expression of manipulated and induced emotions respectively. Users can express happiness without considerable difficulty or effort. However, participants were required to invest approximately twice the workload of happiness to express sadness in both procedures. All elements of the sadness workload had higher scores than those of the happiness workload because sadness requires a more complex facial expression and an additional HR state, which are not essential for recognizing happiness.

The workload for expressing anger was the highest among all emotions. This result reflects the fact that the expression of anger had the longest mean completion time. The other elements of the NASA-TLX were similar in both procedures, except for effort and temporal. The effort and temporal elements were much fewer in the ITE procedure, which means that external stimulation helped the participants to drive the emotion of anger. Hence, these elements are reflected in a relatively low value: the mean completion time for expressing anger, effort, and temporal elements in the NASA-TLX.

Expressing surprise also yielded interesting results. The workload for the emotion of surprise was different in each procedure; it was 2.75 times higher in the ITE procedure. Although other emotions have a similar workload in the ITE procedure than in the IDE produce, surprise alone does not follow this tendency. We infer that this result is due to the characteristics of surprise, as mentioned in the previous section.

## 6. Discussion

Based on our experimental results and qualitative questions, we identified the following two major features when using emotions as inputs:

- **Expression differences:** In addition to explicit and implicit emotional expressions, the emotional expression gap should also be considered an important characteristic. For example, the emotional expression gap between imaginary and actual expressions affects significantly affects the performance and workload for the surprise emotion. Most participants replied that they had difficulty inputting facial expressions in a surprising. This implies that acquired knowledge and actual situations can lead to cognitive dissonance, which can disrupt the use of emotions as a part of the system.
- **Emotional loading time:** Although emotional changes may regulate HR through the nervous system via electrical signals, it takes some time for these changes to stabilize and appear, as demonstrated in Section 5.4. This implies that emotion recognition using physical body condition changes has a higher cost for recognition of emotions such as anger. However, it can be inferred that the same cost will not be incurred for every recognition because of the persistence of emotions. Consequently, a continuous expression of the same emotion may decrease the emotional expression cost by reducing the emotional loading time.
- **Explicit control of implicit signals:** Because human emotional expression performs both implicit and explicit roles, our proposed technique also uses HR variation to capture implicit signals. However, although our methodology guarantees high accuracy as described in Section 5.3, there is a tendency to cause stress to the user. For instance, participants claimed that they were stressed when they purposefully recalled anger. More specifically, they responded that they felt nervous when anger was recognized by the system, in addition to the displeasure associated with anger. However, when these implicit signals were changed by external stimuli (e.g., watching videos), participants showed a faster completion time and reported a lower task workload, resulting in a more natural representation. This implies that different emotion recognition techniques should be applied to both the IDE and ITE, and they cannot complement each other owing to the proportional relationship between the completion time and task workload.
- **The emotional impact of active emotional input on computer-mediated communication:** We have demonstrated that users’ emotions can be utilized as active input to the system through our proposed technique and some of the user study experiments. However, this only means that it is possible from a system perspective, and we cannot predict how it will impact the conversations or overall interactions between users, a topic that goes beyond the scope of this study. For example, according to our qualitative observations, the participants expressed reluctance to deliberately evoke negative emotions, which could potentially affect the flow of the overall conversation or interaction with the system. Therefore, when designing an AEI system, the emotional impact on the user should also be considered in addition to system accuracy and performance time.

## 7. Conclusions

Mobile communication technology has revolutionized the way individuals communicate, but the absence of nonverbal cues in computer-mediated communication can pose challenges in accurately interpreting emotions. In this study, we present a new approach to recognizing emotions as an active input in mobile systems. To address this issue, the proposed approach combines psychological and neuroscientific principles to accurately and comprehensively assess an individual’s emotions for use as active input in mobile systems. The proposed technique uses facial and heart rate information to recognize users’ primary emotions, which can be easily obtained using a front camera and heart rate sensor. The user evaluation results showed that the proposed technique provides a more efficient and accurate way of expressing emotions with average recognition accuracies of 90% and 82% for induced and intended emotional expression, respectively. The proposed technique has the potential to enhance the user experience and provide more personalized and dynamic interactions with mobile systems through the use of active emotional input.

## Figures and Tables

**Table 1:** Technical/Emotional characteristics of emotions.

Prime Emotions	Emotional Characteristics		Technical Constraint
	Facial	HR	
Neutral	Expressionless	Steady	Facial expression conflicts with anger emotion
Happiness	Distinguishable	Decrease	Sufficient with facial expression only
Surprise	Distinguishable	Increase	Sufficient with facial expression only
Sadness	Relatively hard to distinguish	Increase	Not conflict but hard to recognize by facial expression
Anger	Expressionless	Increase	Facial expression conflicts with neutral emotion

**Table 2:** Accuracy of emotion recognition.

	Recognized	Happiness		Sadness		Anger		Surprise		Neutral	
Actual		IDE	ITE	IDE	ITE	IDE	ITE	IDE	ITE	IDE	ITE
Happiness		**0.95**	**0.72**	0	0	0.05	0.18	0	0	0	0.09
Sadness		0	0	**0.86**	**0.77**	0.09	0.07	0	0	0.05	0.13
Anger		0	0	0.09	0.05	**0.90**	**0.80**	0	0	0	0.15
Surprise		0	0	0	0	0.05	0.05	**0.82**	**0.86**	0.14	0.09
Neutral		0	0	0.05	0.05	0	0	0	0	**0.95**	**0.95**

## Data Availability

Not applicable.
